# Issues in Building a Nursing Home Syndromic Surveillance System with Textmining: Longitudinal Observational Study

**DOI:** 10.2196/publichealth.9022

**Published:** 2018-12-13

**Authors:** Tiba Delespierre, Loic Josseran

**Affiliations:** 1 Equipe de recherche (HANDIReSP) UFR des Sciences de la Santé Simone Veil Université de Versailles Saint-Quentin-en-Yvelines et Université Paris-Saclay Montigny-le-Bretonneux France

**Keywords:** Centers for Disease Control and Prevention, nursing homes, syndromic surveillance, pattern recognition, Delphi technique, sentinel surveillance

## Abstract

**Background:**

New nursing homes (NH) data warehouses fed from residents’ medical records allow monitoring the health of elderly population on a daily basis. Elsewhere, syndromic surveillance has already shown that professional data can be used for public health (PH) surveillance but not during a long-term follow-up of the same cohort.

**Objective:**

This study aimed to build and assess a national ecological NH PH surveillance system (SS).

**Methods:**

Using a national network of 126 NH, we built a residents’ cohort, extracted medical and personal data from their electronic health records, and transmitted them through the internet to a national server almost in real time. After recording sociodemographic, autonomic and syndromic information, a set of 26 syndromes was defined using pattern matching with the standard query language-LIKE operator and a Delphi-like technique, between November 2010 and June 2016. We used early aberration reporting system (EARS) and Bayes surveillance algorithms of the R surveillance package (Höhle) to assess our influenza and acute gastroenteritis (AGE) syndromic data against the Sentinelles network data, French epidemics gold standard, following Centers for Disease Control and Prevention surveillance system assessment guidelines.

**Results:**

By extracting all sociodemographic residents’ data, a cohort of 41,061 senior citizens was built. EARS_C3 algorithm on NH influenza and AGE syndromic data gave sensitivities of 0.482 and 0.539 and specificities of 0.844 and 0.952, respectively, over a 6-year period, forecasting the last influenza outbreak by catching early flu signals. In addition, assessment of influenza and AGE syndromic data quality showed precisions of 0.98 and 0.96 during last season epidemic weeks’ peaks (weeks 03-2017 and 01-2017) and precisions of 0.95 and 0.92 during last summer epidemic weeks’ low (week 33-2016).

**Conclusions:**

This study confirmed that using syndromic information gives a good opportunity to develop a genuine French national PH SS dedicated to senior citizens. Access to senior citizens’ free-text validated health data on influenza and AGE responds to a PH issue for the surveillance of this fragile population. This database will also make possible new ecological research on other subjects that will improve prevention, care, and rapid response when facing health threats.

## Introduction

### Background

Population in developed countries is aging [1], and the French population follows this trend. In France, by 2050, 22.3 million people will be aged over 65 years compared with 12.6 million in 2005, an increase of 80% in 45 years. Between 2013 and 2050, the senior population will grow more than the population as a whole. Similarly, life expectancy at birth in France, one of the highest in the world, is projected to surpass 86 years for men and 90 for women [2].

This increase will then have to be anticipated and will affect care and related costs [3]. It is, therefore, essential to improve our knowledge of this senescence process to help prevent increase in pathologies, and improve quality of life at extreme ages.

In spite of this major expected evolution of population, ecological research on this aged population is still limited [4]. Case or ad hoc studies do not consider individual variability and cannot analyze health issues as a whole. Data then need to be recorded for quite a long time and on a daily basis, helping to address this lack of knowledge. This has to be done in a natural way, in a professional environment with caregivers and medical staff [5].

As until now, follow-up studies on senior citizens were conducted using cohorts that were costly to set up and follow [6-8]. Data are occasionally stored, even if the follow-up is long and based on auto-questionnaires or planned interviews with health professionals. This approach does not allow describing in detail the daily life of this population and storing health evolutions throughout the residents’ whole stays.

### New Data

On the contrary, nursing homes (NH) offer this possibility of tracking and recording them daily as health professionals feed these information for their proper use and, this time, without any memory bias [9]. These new data as well as their uses suggest innovative approaches to improve health knowledge.

Korian (Paris, France) as the first private NH European group has these kinds of data. This enterprise holds 290 NH and approximately 3.92% (290/7394) [10] of the French NH network, distributed all over the country, mostly in urban areas (see Multimedia Appendix 1). A professional data warehouse (DWH) set up in 2010 hosts half the company’s French residents’ population data. Their health follow-ups are recorded daily from 126 NH. For every new resident admitted in one of the NH, a personal electronic resident medical file (PERMF) is set up. Data are then collected at various times: at admission (admission date, medical history, marital status, birth date, tastes, and habits), on a daily basis (new pathologies, chronic disease evolution, date of death, and drug prescriptions), or just after specific medical or health care professional visits. Items include diagnosis, outcomes, as well as sociodemographic information.

### Objectives

Elsewhere and a little earlier, during the early 2000s, surveillance systems (SS) [11-19] showed that professional data could also be used for health and alert surveillance [20-26]. Here, professional data use for SS was only done using point data analysis (going to the emergency, 911, and Web queries) [19,25,26] and not during a long-term follow-up of the same people, and even more, not dedicated to senior citizens.

As we have just seen, data gathered by different NH professionals offer the opportunity of following the residents’ situations on the flow and on a daily basis and, through this process, of building syndromic surveillance data. The objective of this study was to build and assess an influenza and acute gastroenteritis national ecological NH public health (PH) SS describing and validating the Base du Bien Vieillir (BBV), that is, Aging Well database architecture. Thus, through a new health data building paradigm, we engineered an NH syndromic surveillance system (SSS) based on already validated criteria [11,13], hopefully opening the way to new research and knowledge about the senescence process.

## Methods

### Data Collection

All data are transmitted from 126 NH in real time to a national server using the group intranet. Records collected from the PERMF server are anonymized (see Multimedia Appendix 2) when sent to the BBV server, keeping track of every resident even when moving from one NH to another. After this first step, BBV is built through an extract, transform, and load (ETL) process of health and sociodemographic data. (see Multimedia Appendix 3 for details). Following this second step, all residents have two types of data:

1. Gender, age, and GIR (Groupe ISO Ressources; english: Group International Standardization Organization Resource Group), a French autonomy-level rating indexed to government benefits [27-32] at the NH entry
2. Daily care information fed on the flow by the caregivers and the medical staff, whenever deemed useful, that is, their syndromic information and, finally, hospitalizations and death

At the same time, every Sunday, all residents’ daily care information is aggregated to count the weekly number of syndromes per NH.

By extracting data for all residents of the PERMRF database from its inception, from November 1, 2010, to mid-February 2017, and adding every new resident entering one of the NH networks every week, a *one-week moving* cohort of residents followed during their entire NH life course was built opening the way for our SSS. Even if most residents of this cohort were followed during their entire NH life course, syndromic data could be left-truncated for people whose data were entered before the inception of the information system (IS) or right-truncated for people whose data were entered later.

At the IS core, the data transmissions table containing key information about the residents’ care was fed on a daily basis. Data take the form of big size character fields (of up to 4000 characters). Extracting these and using residents and NHs’ indexes and data transmissions dates (see Multimedia Appendix 3 for a complete example), we were able to track all residents through two dimensions. Over time—every day with syndromic data—and space—every NH with syndromic data—with queries and text mining, building their syndromic life course, beginning at their date of entry and ending with their last available data transmission or death. The BBV then has two nested time frames: by day for every resident and by week for every NH.

### Ethics Approval and Consent to Participate

The use of this database in the frame of epidemiological studies has been authorized by the French National Commission for Data protection and Liberties. The Institut du Bien Vieillir, which became the Foundation Korian of Well Ageing, filed a declaration of conformity to a baseline methodology, which received an agreement number in March 2017: 2.041.050, in accordance with the Act n 78–17 of January 6, 1978 on Data Processing, Data Files, and Individual Liberties. All residents are informed at their NH entry about their electronic health record (EHR) and their right to oppose its use. Although the primary purpose of this medical research was to generate new knowledge, this goal did not take precedence over the rights and interests of the NH residents. All the new generated information was extracted from already existing data and was deidentified and anonymized when necessary to protect their health and rights.

### Building the Acute Respiratory Infection and Influenza-Like Illness and Acute Gastroenteritis Syndromes

With a multistep learning and text mining (MSL-TM) process (see Multimedia Appendix 3 for the 4-phase process) of the data transmissions file similar to what was experimented in the study by Cohen at al [33], using problems’ list logic [34-36] and pattern matching with the SQL-like operator [37], 24 syndromes were implemented [38-49], following the SurSaUD (Sanitary Surveillance of Urgencies and Deaths) SSS method [16]. Starting with acute respiratory infection and influenza-like illness (ARI-ILI) and acute gastroenteritis (AGE) syndromes (see Multimedia Appendix 3 for 2 examples and the syndromes’ list), extracting directly hospitalizations and deaths, this NH IS kept for every resident, every day, in every NH, from none to 26 daily syndromes whenever appropriate (see Multimedia Appendix 3 again for full details of the whole process [50-61]).

### The Surveillance Tools Framework

Syndromic Systems attempt to detect outbreaks through statistical analysis of aggregated cases data to improve on competent clinicians in detecting early-stage or small outbreaks [62]. It focuses on data collected before clinical diagnosis or laboratory confirmation [63]. Statistical laws are then defined to give an answer to the question “knowing the average number of expected events during a period of time, what is the probability to observe the current situation?” [62].

**Figure 1:**
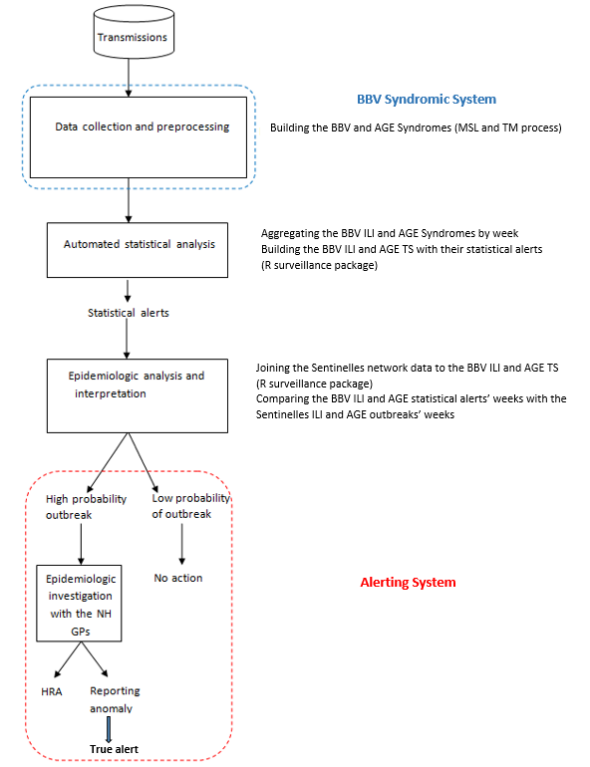
The nursing homes acute respiratory infections and influenza-like illness and acute gastroenteritis surveillance tools framework. BBV: Base du Bien Vieillir (ageing well database); ILI: influenza-like Illness; AGE: acute gastroenteritis; MSL: multi-step learning; TM: text mining; TS: time series; GP: general practioner; HRA: Health Regional Agencies.

The SSS generation was designed using a *Pentaho* extraction platform for all the ETL processes [64] and is described in Figure 1. It follows the Centers for Disease Control and Prevention (CDC) Working Group recommendations [11,13].

The whole process was done in 4 steps: first, the ILI and AGE syndromes built through the MSL-TM process [65]; second, the weekly ILI and AGE syndromic data aggregation and the time series (TS) generation with their statistical alerts using the R *surveillance* package [66,67]; third, the Sentinelles data joining, the ARI-ILI and AGE French surveillance gold standard [68]; and finally, the alerting system interfacing the *surveillance* package [66,67] statistical alerts with the NH general practitioners (GPs) coordinators signals, eventually reporting to the Health Regional Agencies (HRA).

It is only after the final step that epidemiologists in the national public health agency’s regional units (HRA in Figure 1) are asked to choose an alert level for the regions they are in charge of: non-epidemic, pre/post epidemic, or epidemic [68]. A public health alert will then be defined as such by the public health agency Santé publique France (SPF) after every signal has been verified and validated [69] (for further details, see Multimedia Appendix 4 [22,67-76]). Relevant information for French epidemiologists since January 2016 at a regional level includes the Sentinelles (2.1% of French private GP) as well as the OSCOUR (Organization of the COordinated Surveillance of Urgencies; 88% of French hospital emergency departments make up the Coordinated Health Surveillance of Emergency Department network) and SurSaUD (95% of French emergency GP consultations) data but also local specific surveillance data such as NH ARI clusters’ surveillance.

### Syndromic Data Analysis

#### Data Flow Buildup and Stabilization

**Figure 2:**
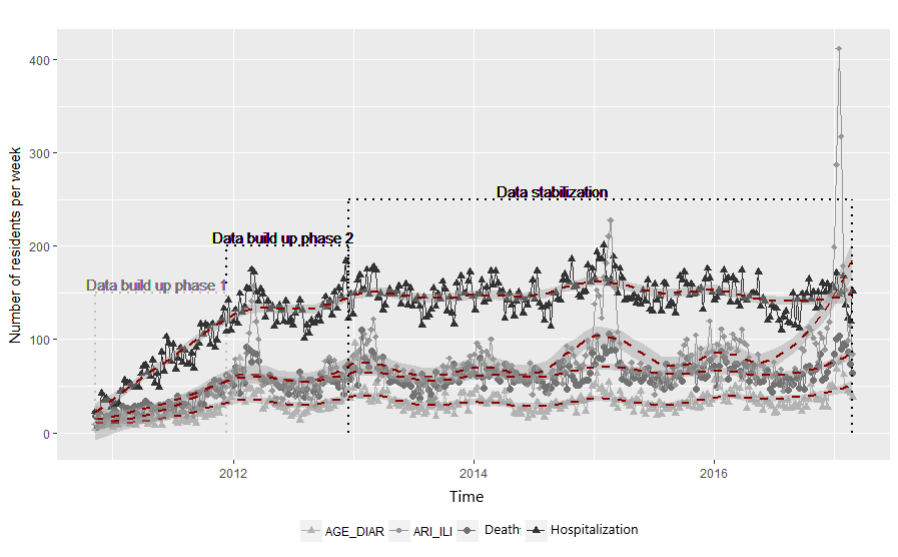
Acute gastroenteritis, acute respiratory infections, deaths and hospitalizations data flow buildup and stabilization in 11 regions covering France between November 1, 2010, and February 26, 2017. AGE-DIAR: acute gastroenteritis and diarrhea; ARI-ILI: acute respiratory infection and influenza-like illness.

As explained above, we computed weekly counts of ILI and AGE cases as well as hospitalizations and deaths as with *Sentinelles*, avoiding week and weekend days’ heterogeneity [77]. Then, with the *ggplot* function of the *ggplot2* R package [78] used with local regression curves fitted to the NH data (Figure 2) [79], we were able to track yearly tendencies as well as inconsistent data not reflecting the seasonal spikes during winter.

Assessing the syndromic data flow over time, by computing the summary statistics of deaths, hospitalizations, ARI-ILI and AGE weekly syndromes’ counts during the 3 periods (ie, from November 1, 2010, to November 1, 2011; then from November 1, 2011, to November 1, 2012; and finally from November 1, 2010, to February 26, 2017), we chose to exclude the first year’s data from this analysis (see Table 1).

**Table 1:** Assessing the Base du Bien Vieillir (BBV) four syndrome’s weekly counts.

Period	Minimum	Q1	Median	Mean	Q3	Maximum
Phase 1	4	16	24.5	33.45	42.25	118
Phase 2	13	40.5	55	70.92	109.25	184
Stabilization	15	48	67	82.72	124	412

#### Building the Acute Respiratory Infection and Influenza-Like Illness and Acute Gastroenteritis Time Series

The ARI-ILI and AGE TS were built by aggregating all NH weekly syndromes counts. The choice of a statistical method to analyze these then rested on definitions of statistical alerts, adapting the BBV SSS data to fit French public health infrastructure and available SS data sources [80], here data of the ARI-ILI and AGE Sentinelles network [22]. Although the Sentinelles network used the Serfling method [70,71] relying on disease incidence levels of preceding years, we used the CDC steady favorite, the CUSUM (cumulative sums) methods, not drawing on data from preceding years but just from preceding weeks and 1 recent method, the Bayes method allowing fine tuning [72]. For further details, see Multimedia Appendix 4.

#### Quality-Precision

Following the process described in the Building the Acute Respiratory Infection and Influenza-Like Illness and Acute Gastroenteritis Time Series subsection, the whole procedure was reviewed over 3 weeks of data transmissions: one in mid-August 2016 when there was no epidemic and two in January 2017 at the ILI and AGE epidemics weeks’ peaks, respectively, according to the Sentinelles network [81], computing the percentage of miscoded ARI-ILI and AGE syndromes among the extracted data transmissions defined as such [26].

#### Stability

The idea here was to check the syndromic flow stability in quantity (the syndromes counts) and quality (several different recurring syndromes) during the complete period and for all 126 NH, computing the weekly syndromes frequencies for every NH.

The syndromic data flow stability was traced by designing 3 chronic diseases and 1 often-chronic ailment indexes [82] built as follows: whenever a resident had diabetes or a cardiovascular problem or depression or fell, the resident’s transmission date and syndromic event type were set apart. Then, a similar event during a 200-day period after this resident’s syndromic event was searched for, defining 4 syndromic ratios for the 6 years from the year 2011 up to February 27, 2017. For further details, see Multimedia Appendix 5.

#### Flexibility-Timeliness-Representativeness-Usefulness

Adaptability and reactiveness of the system were evaluated during the outbreak and routine periods according to the CDC surveillance systems guidelines [18,15,26]. Representativeness, completeness, and usefulness were assessed using the distribution description of ILI cases by time and origin during this last flu season, as well as by rating sex, age, and GIR at entry and age at illness missing data [13].

#### Surveillance Algorithms’ Quality

All surveillance methods involve building first, time series with the weekly number of cases and second, statistical indicators used as thresholds. Here the 4 algorithms were compared by using the *algo.quality* function for Bayes [72] and rebuilding it for the early aberration reporting system (EARS) algorithms. This quality is defined by 4 numbers—the number of true positive (TP), false positive (FP), true negative (TN), false negative (FN)—and 4 criteria—the *sensitivity* [83] sometimes called recall [84] as the ratio of epidemic weeks correctly identified; the *specificity*, as the ratio of nonepidemic weeks correctly identified; the Euclidean distance between the perfect method with *specificity=sensitivity=1* and ours (*distance=((1-specificity)*^*2*^*+(sensitivity-1)*^*2*^*)*)^1/2^; and finally, the *precision* or *positive predictive value* (PPV), as the ratio of epidemic weeks correctly identified among the weeks defined as epidemic (with statistical alarm) [13].

## Results

### The Cohort

As explained in the Data Collection subsection, by extracting all residents already there on November 1, 2010, and then by adding those entered every week in one of the 126 NH, a cohort of 41,061 residents (Figure 2) was built with 12,983 men (31.61%, 12,983/41,061) of mean age 84.33 years and 28,083 women (68.39 % 28,083/41,061) of mean age 85.82 years.

#### The Acute Respiratory Infection and Influenza-Like Illness and Acute Gastroenteritis Syndromes and the Surveillance Inside Korian

As described in Building the Acute Respiratory Infection and Influenza-Like Illness and Acute Gastroenteritis Syndromes subsection, the BBV syndromic algorithm extracted all ARI-ILI and AGE cases in addition to hospitalizations and deaths, every week, from November 1, 2010, to mid-February 2017 and built the 4 TS. Using the BBV ARI-ILI and AGE syndromic TS, we were able to track the last flu season (winter 2016-2017) early on, even before the epidemic and compare our syndromic counts with the Korian GPs’ number of cases. The first ones were usually much greater than the second ones, as several syndromic cases could identify the same resident over time, but both of them were always strongly correlated.

### Syndromic Data Analysis

#### Checking the Data Flow During Time

We managed to highlight 3 different phases in the NH data flow as shown in Figure 2 and Table 1 with two buildup phases during the first 2 years of the IS implementation. As seen below, between the first and the second year, the median and mean weekly syndromes’ counts more than doubled. For that reason, we excluded the data of the first year from the syndromic data analysis.

#### The Acute Respiratory Infection and Influenza-Like Illness and Acute Gastroenteritis Time Series

**Figure 3:**
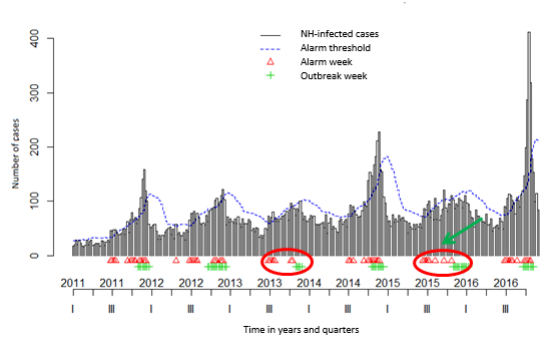
The influenza-like illness (ILI) Base du Bien Vieillir time series (TS) using the Bayes’ alarm algorithm with 12 weeks upstream and the ILI Sentinelles outbreaks. NH: nursing homes. Green ellipses highlight a nice overlapping of alarm and Sentinelles’ network weeks or when the algorithm seems better, whereas red ellipses when this is not the case.

**Figure 4:**
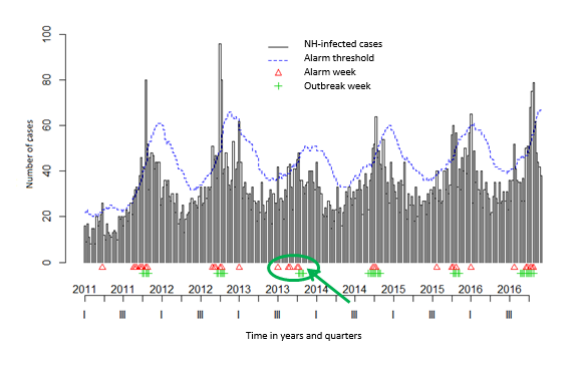
The acute gastroenteritis (AGE) Base du Bien Vieillir (BBV) time series using the Bayes’ alarm algorithm with 12 weeks upstream and the AGE Sentinelles outbreaks. NH: nursing homes. Green ellipses highlight a nice overlapping of alarm and Sentinelles’ network weeks or when the algorithm seems better, whereas red ellipses when this is not the case.

**Figure 5:**
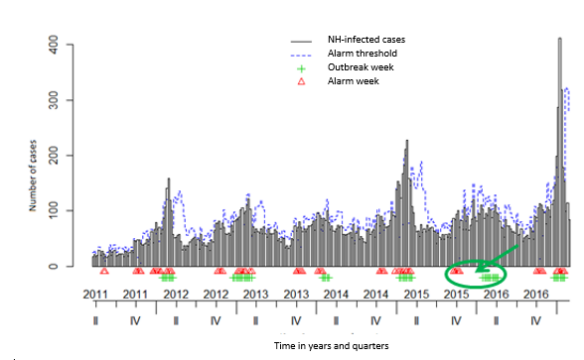
The influenza-like illness (ILI) Base du Bien Vieillir (BBV) time series using the EARS_C3’s alarm algorithm with 12 weeks upstream and the ILI Sentinelles outbreaks. NH: nursing homes. Green ellipses highlight a nice overlapping of alarm and Sentinelles’ network weeks or when the algorithm seems better, whereas red ellipses when this is not the case.

**Figure 6:**
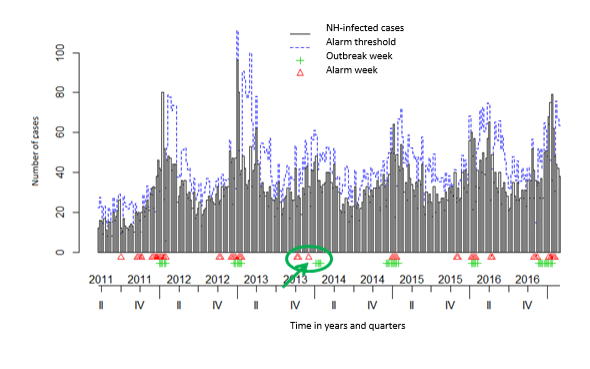
The acute gastroenteritis (AGE) Base du Bien Vieillir (BBV) time series using the EARS_C3’s alarm algorithm with 12 weeks upstream and the AGE Sentinelles outbreaks. NH: nursing homes. Green ellipses highlight a nice overlapping of alarm and Sentinelles’ network weeks or when the algorithm seems better, whereas red ellipses when this is not the case.

In the *surveillance* package, both Bayes’ (see Figures 3 and 4) and EARS_C3s’ algorithms with alpha=.025 (see Figures 5 and 6) used the 12 former ARI_ILI (Figures 3 and 5) and AGE (Figures 4 and 6) NH weeks’ counts to define *alarm weeks* (red triangles). *Outbreak weeks* (green vertical lines) were defined according to the ILI and AGE Sentinelles data during the same period (from January 1, 2011, to January 16, 2011). Finally, the blue dotted lines were the upper limits at which alarms were triggered with both algorithms.

Senior citizens suffer much more of either ARI-ILI or AGE than the general population all year long and even in summer. This often results mechanically in triggering statistical alerts long before the general population epidemics. It can be seen in Figures 3 and 5 for ARI_ILI and Figures 4 and 6 for AGE where the red triangles (the SSS alarm weeks) appear always before the green bars (the Sentinelles’ network outbreak weeks). It is especially true for ARI-ILI during the 2013 to 2014 and 2015 to 2016 winters and for AGE during the 2013 to 2014 winter with both algorithms.

#### Quality-Precision

To compute the percentage of miscoded ARI-*ILI* and *AGE* syndromes, all ARI-ILI and AGE syndromic data were extracted during 3 weeks: one in mid-August 2016, the 33rd week (third column) when there was neither flu nor AGE epidemic, and two in January 2017, the third and first weeks, at the ILI and AGE epidemics weeks’ peaks, according to the Sentinelles network (second column). Then, each ILI and AGE syndromic data transmission was examined, rating it as correct, adding to TP, or incorrect, adding to FP (see Table 2).

The precision was best during epidemic weeks’ peaks: 98% and 96%, respectively, as ILI and AGE versus 95% and 92% in summer, and there were very little FP. For example, for ILI FP, *“* his son has flu,” “emergencies overloaded with flu cases,” “no flu symptoms,” “could they take care of my girl who has flu?”, and finally, “serrure dégrippée,” which means *unjammed* in French but has the same word stem “gripp” as flu, were excluded. We had already excluded the word *“* grippé*”* in this context, which means *jammed* for a lock. In addition, by checking the flu cases, we found 67 flu tests mentions using nasal swabs, adding 21% new cases. For AGE FP, “vomited without diarrhea” (2 times, as both words are needed to classify as an AGE syndrome) and “diarrhea protocol if fever” were excluded.

**Table 2:** Assessing Base du Bien Vieillir (BBV) influenza-like illness (ILI) and acute gastroenteritis (AGE) syndromes data transmissions precision during 3 periods: last summer (2016: 33rd week), the Last ILI (2017: 3rd week) and (2017: first week) epidemics weeks’ peaks.

Disease	Epidemic period	Week of study	Residents with syndromes, n	Residents with syndromes x transmission days, n	Residents with influenza-like illness or acute gastroenteritis, n	False positive	True positive	Precision^a^ or positive predictive value
ILI	No	sem33-2016	5399	10,631	56	3	53	0.95
AGE	No	sem33-2016	5399	10,631	26	2	24	0.92
ILI	Yes	sem03-2017	6013	12,215	318	5	313	0.98
AGE	Yes	sem01-2017	5862	11,933	68	3	65	0.96

^a^Precision=TP/(TP+FP).

#### Stability

As detailed in this section, the data transmission stability was evaluated by studying the weekly syndromes’ frequencies for every NH from November 1, 2010, as well as the ratio of weeks with syndromic data transmissions:

1. The weekly 26 syndromes frequencies averaged over the number of NH (126) ranged from 21.45 to 180.57 (mean=88.5, SD=22.8).
2. The ratio of weeks of data transmissions per NH was built by computing the number of data transmissions weeks versus the data transmissions weeks span and ranged from 89% to 100% (mean=100%, SD=1%). Only 1 NH had a ratio of less than 95%.

Finally, the syndromic data flow over time studied with 3 chronic illnesses and falls syndromes distributions showed great stability (see Multimedia Appendix 5 for further details).

#### Flexibility-Timeliness-Representativeness-Usefulness

Table 2 showed the flexibility and reactiveness of our syndromic system during epidemic periods, following at the same time the increasing number of cases, going from a weekly population of 5399 to 6015 without losing any precision, from 95% to 98%. As shown this winter, by using the NH indexes and regions, the flu epidemic was followed geographically, week after week, starting from the beginning, detecting where the epidemic was most intense, tracking the most severe cases and related hospitalizations and deaths.

Great geographic heterogeneity was detected between regions in terms of ratios of infected; the Rhone valley (Rhone Alpes) and south (Sud) being the most afflicted with 24% and 19% and the southeast (Sud-Est) being the least afflicted with only 9%. There was also great variability in terms of population characteristics, for example, those from the southwest were the oldest afflicted at a mean age of 89.7 years, whereas the southern residents were the youngest at 87.6 years, 2 years and a month difference being quite a lot given the mean duration of residents’ stay. Finally, among 1800 residents with a flu transmission, only 1 had no personal data.

#### Surveillance Algorithms’ Quality

The 3 surveillance package EARS’s algorithms as well as Bayes’ algorithm were compared for both diseases. For every algorithm, the CI level defines the threshold used to trigger statistical alarms. We used a CI level of 0.001 for EARS_C*i*, *i=1, 2, 3* and also a CI level of 0.025 for EARS_C3 and Bayes’ algorithms. 0.001 is the EARS_C3 default level, whereas 0.025 is the Bayes’ algorithm default level (see the algorithm quality in Table 3). The EARS_C3 with alpha=0.025 gave the best results for both diseases (in italics in Table 3). Nevertheless, the Bayes’ algorithm seemed better to define alarm weeks when epidemics were less intense as for ILI 2015 to 2016 and AGE 2013 to 2014 seasons, as there were fewer lag weeks between the Bayes’ alarm weeks and the Sentinelles outbreak weeks (green arrows were added in Figures 3-6 to highlight this trend).

Either with ILI or AGE TS, mostly coherence between NH data and the Sentinelles data could be witnessed. In addition, only 12 weeks of data (see Table 3) were needed to detect outbreaks, most of the time several weeks ahead of Sentinelles’ outbreaks. This was especially true for the last flu season (winter 2016 to 2017 in Figures 3 and 4).

**Table 3:** Comparing the surveillance algorithms’ quality on Base du Bien Vieillir (BBV) influenza-like illness (ILI) and acute gastroenteritis (AGE) time series with ILI and AGE Sentinelles’ outbreaks detection, by using early aberration reporting system (EARS).

Disease and algorithm		Number of weeks	True positive	False positive	True negative	False negative	Sensitivity or recall	Specificity	Distance^a^	Precision^b^ or positive predictive value
**Influenza-like illness**										
	Bayes^c^	12	22	44	210	32	0.407	0.827	0.617	0.333
	EARS_C1^d^	7	6	12	248	48	0.111	0.954	0.890	0.333
	EARS_C2^d^	9	13	26	232	41	0.241	0.899	0.766	0.333
	EARS_C3^d^	12	19	31	225	35	0.352	0.879	0.659	0.380
	*EARS_C3* ^c^	*12*	*26*	*40*	*216*	*28*	*0.482*	*0.844*	*0.542*	*0.394*
**Acute gastroenteritis**										
	Bayes^c^	12	16	18	251	23	0.410	0.933	0.594	0.471
	EARS_C3^d^	12	16	13	258	23	0.410	0.952	0.607	0.592
	*EARS_C3* ^c^	*12*	*21*	*26*	*245*	*18*	*0.539*	*0.904*	*0.471*	*0.447*

^a^Distance=sqrt ((1-spec)^2^ + (sens-1)^2^) is the Euclidean distance of (specificity, sensibility) from (1, 1).

^b^Precision=TP/ (TP+FP) is the true positives ratio among the positives.

^c^CI with alpha=.025. Italicization indicates the best results, and therefore, the best method to detect both ILI and AGE.

^d^CI with alpha=.001.

## Discussion

### Principal Findings

We built and assessed a national ecological NH PH SS dedicated to senior citizens. By using a national network of 126 NH and extracting all sociodemographic as well as daily medical data from EHRs, a cohort of 41,061 residents was built. Through textual analysis of clinical narratives (CNs), we implemented ARI_ILI and AGE syndromes. We also engineered related TS by computing weekly head counts. Alarms with EARS_C3 and Bayes algorithms on these, over a 6-year period, allowed us to forecast the 2016 to 2017 influenza outbreak by more than 2 weeks; as can be seen in Figure 5, our statistical alarms were triggered in December, whereas the influenza epidemic according to SPF started only in January.

With just 4 tables, this IS of a new kind showed that it is possible to follow almost every resident every day, where he or she is, during his or her entire NH life, hopefully selecting most of his or her ARI-ILI and AGE health events, from NH entry until death or exit. Furthermore, each relevant syndrome is defined by 2 syndromic representations: either a simple additive syndromic image, that is, its 4 Boolean [65] syndromic components allowing whatever filtering, or its literal expression for further textual analysis or in-depth health questioning. By this whole process, free textual information extracted from CN was shaped into numerical data for further statistical or machine learning analysis.

In this study, we engineered a real NH SSS on qualitative data, offering immediate accessibility without adding any extra work to medical staff [11]. By using SQL-like pattern matching [37] and Delphi-like experts’ consensus [57,58] on the data transmissions file, we followed last season ARI-ILI and AGE epidemics and found almost in real time that the flu dramatically reached NH residents, tracking them geographically and timely, searching for flu-related hospitalizations and deaths. Preventing disruptions of medical tasks and medical and paramedical staff turnover by predicting even 1 or 2 weeks ahead, the epidemic intensity could greatly improve the NH human resources management over time and help to prevent sanitary disasters by strengthening hygiene measures, for example.

As explained in [12], early detection of outbreaks can be achieved in 3 ways: first, by prompt recognition and reporting of disease case reports. Here, we could find most of flu and AGE cases by syndromic descriptions fed in the data transmissions table. Second, by improving the ability to recognize patterns indicative of a possible outbreak early in its course, using analytic tools, counting syndromes by NH, and building time series with the surveillance package. Third, by exploiting data that can signify an outbreak earlier in its course. More specifically, adding hospitalizations and deaths syndromes to the ARI_ILI, AGE syndromes allowed us to assess the flu and AGE outbreaks intensities as well as their severities long before the French health authorities this last season and follow precisely and locally the residents’ syndromic population because of the NH and residents’ indexes.

This framework with its 3 components, wholly described in Figure 1, has shown its efficacy as a public health SS for early detection of outbreaks. By bringing to light new data not available elsewhere when needed, this SSS improves NH ARI-ILI epidemics’ knowledge. Its tools’ efficacy could even be quantified by assessing syndromes’ precision, stability, flexibility, timeliness, representativeness, and finally algorithms’ quality [12,66].

For the AGE data, even with lots of cases, a good correlation could be found for every winter season between the NH alarm weeks and Sentinelles outbreak weeks (as shown in the last row in Table 3 by the small distance value of 0.471). The first ones almost always precede the latter by several weeks, except for the 2014-2015 winter where the AGE epidemic essentially reached senior citizens in NH [85]. During last winter, the AGE outbreak started at the same time as in other NH in France.

### Limitations

This SSS using mostly the transmissions’ qualitative data is neither exhaustive as some syndromes may still not be described in the SSS nor complete, as medical staff may not have fed all syndromic information on some day for whatever reason. So, ILI and AGE syndromic data recall, what proportion of cases in classes were correctly assigned to their classes [65], could not be assessed. At this moment, the syndromic information depends essentially on the medical staffs’ available time and dedication to feed the system as shown in the Results section Syndromic Data Analysis subsection Stability subsubsection, where 1 NH had a ratio of data transmissions weeks of 89%, with 293 weeks of data transmission over a total span of 329 weeks.

As soon as the cold season begins, elderly people may get a respiratory syncytial virus (RSV), similar to very young children. In fact, RSV is a common cause of acute respiratory illness in older adults as the risk of serious respiratory infection increases with age [86,87]. Usually, RSV spreads quickly just before flu or at the same time and is largely indistinguishable from influenza based on clinical presentation alone [50,51,86]. It is rather a recurring problem in older adults causing 2% to 5% of adult community-acquired pneumonia [88]. Triggering an alarm even for RSV would allow to quickly organize care for the residents*.*

Then, by following our syndromic ARI_ILI data, 2 trends could be traced, one starting in early November, maybe the RSV, followed by another one later, starting usually in December as this year or later as last year. Depending on the flu epidemic characteristics and as ARI, ILI, and RSV could not be distinguished in our text mining algorithm, a flu threshold could be detected whenever appropriate or several weeks ahead. As can be seen, during the 2013-2014 and 2015-2016 winters, between the first alarm weeks and the outbreak weeks, quite long times elapsed [89,90], but as not really reaching elderly people, there was not something clear to find. However, during the 2014-2015 and 2016-2017 winters, we found a much better correlation between the two, the first ones, probably because of RSV, always preceding the latter by approximately 8 weeks (Figures 3 and 4), thus often triggering alarms before those of the Sentinelles network.

At the same time, we found proportionately much more ILI new cases with our SSS than with the Sentinelles network, especially for this last influenza season (see the last ARI-ILI surge at the beginning of 2017 in Figures 3 and 4). As a type A influenza virus, it reached people older than 75 years much more than the rest of the French population [81]. Then, as soon as clusters of NH ARI cases appeared, many flu tests had to be done to label residents as flu positive or negative. Moreover, even as some tests were negative, they derived from the flu epidemic health protocol and were mandatory to HRA hygienic safety measures [91-95], increasing the number of cases still more.

Nevertheless, as detailed above, fewer lag weeks were found with the Bayes’ algorithm and even an overlap of alarm weeks and outbreak weeks for the ILI 2015-2016 (Figure 3) and AGE 2013-2014 (Figure 5) epidemics and nothing like that with the EARS_C3 algorithm (Figures 4 and 6). We could try in the following years to mix both algorithms as done in the study by Baroukh [96] for Salmonella and decide triggering an alarm whenever 1 of the 2 algorithms reaches its alarm threshold, probably improving both sensitivity and specificity. Alternatively, as in the new MASS (Module for the Analysis of SurSaUD and Sentinelles’ data) system [68] designed by SPF, we could combine 3 statistical methods and 3 different data sources, used since January 2016 to define the public health alerts.

Finally, the epidemiologic analysis and interpretation steps (Figure 1) were not fully automated. Some work still needs to be done, especially the whole Sentinelles data extraction process. Some similar job was done before on another project [96,97].

### Conclusions

Outbreak alerts are more reliable when systems focus on specific syndromes that reflect high-probability events such as influenza [62], as could be seen in this real-life experiment. However, there is always room for improvement, as the aggregation of ARI and ILI as well as RSV constraint shows. Nevertheless, this IS gives already a rich and detailed *syndromic* image of these residents. Moreover, as syndromes are modular and the *Pentaho* platform [64] allows extraction from different data silos, it will be possible to add new syndromes, maybe RSV, whenever needed and to adapt them to the new IS that is twice as big and due next year.

This study follows another work on CN using textual analysis and clearing the way for this syndromic health IS design [98]. Tracking flu and AGE epidemics seasons almost in real time and following their impact especially during this last year acute flu season has helped to show the usefulness of this SSS. In addition, the (November 2010-June 2016) syndromic data were used to build ARI_ILI and AGE algorithms, and nothing had to be added or retrieved to follow these last season epidemics’ trends, so these algorithms exhibited flexibility, adaptability, stability, and timeliness.

This study highlights some differences between the NH residents’ population and the general population, which hampers a better correspondence between NH alarm weeks and Sentinelles outbreak weeks. The main challenges here are extending the syndromic IS, improving the syndromes descriptions, as well as better taking into account NH residents’ distinctiveness. Monitoring flu and AGE using the BBV IS could give way to a real SS for all senior citizens in France. For example, there are incoming discussions between Korian and HRA about targeting RSV besides flu and handling what differentiates them.

Korian NHs are already working with HRA at a local level, exchanging clinical data with them whenever outbreaks are detected. This data sharing could then be extended with syndromic data integration, resulting in HRA reactiveness improvement [99]. Indeed, syndromic data are always available before, even if less precise. NH residents as a whole are a frail and captive population functioning as an ever-increasing reservoir for any contagious illness [100,101]. It is then essential to be able to prevent with all possible disposable tools any health catastrophe in the near future.

This syndromic IS offers a real opportunity for finding new ways to seniors’ functioning modelization and opens, hopefully, the path toward specific clinical hypotheses formulation. Other works included studying the use of this IS applied to other public health problems such as frequent falls or falls with casualties [102] but also working toward a better life ending with cancer [103]. Ultimately, the aims are removing all preventable deaths and improving the residents’ end of life with more autonomy, less pain, and an improved quality of life, translating this new knowledge into health benefits for seniors everywhere.

## Appendix

Multimedia Appendix 1 The Korian nursing home network in France.

Multimedia Appendix 2 The anonymization process.

Multimedia Appendix 3 The Base du Bien Vieillir (BBV) acute respiratory infection and influenza-like illness and acute gastroenteritis syndromic information building process in 4 phases: the 4 BBV tables, 2 syndromic examples, and the BBV 26 syndromes list.

Multimedia Appendix 4 How public health alerts are defined: statistical alerts according to the Sentinelles network; true alerts according to the MASS system; and how the surveillance package works.

Multimedia Appendix 5 Details about syndromic data-flow stability rating syndromic data flow stability over time with 4 syndromes frequencies: diabetes, cardiovascular problems, depression, and frequent falls.

## Abbreviations

AGE: acute gastroenteritis
ARI: acute respiratory infection
ARI-ILI: acute respiratory infection and influenza-like illness
BBV: Base du Bien Vieillir
CDC: Centers for Disease Control and Prevention
CN: clinical narrative
CUSUM: cumulative sums
EARS: early aberration reporting system
EHR: electronic health record
ETL: extract, transform, and load
FN: false negative
FP: false positive
GIR: groupe ISO ressources - ISO resources group
GP: general practitioner
HRA: Health Regional Agencies
ILI: influenza-like illness
IS: information system
MSL-TM: multistep learning and text mining
NH: nursing homes
PERMF: personal electronic resident medical file
PH: public health
PPV: positive predictive value
RSV: respiratory syncytial virus
SPF: Santé publique France
SQL: standard query language
SS: surveillance system
SSS: syndromic surveillance system
SurSaUD: Sanitary Surveillance of Urgencies and Deaths
TN: true negative
TP: true positive
TS: time series
