# Supplementary figures and images for: Issues in Building a Nursing Home Syndromic Surveillance System with Textmining: Longitudinal Observational Study
Source: JMIR Public Health Surveill. 2018 Dec 13;4(4):e69. doi: 10.2196/publichealth.9022 (PMC6315244; doi:10.2196/publichealth.9022)

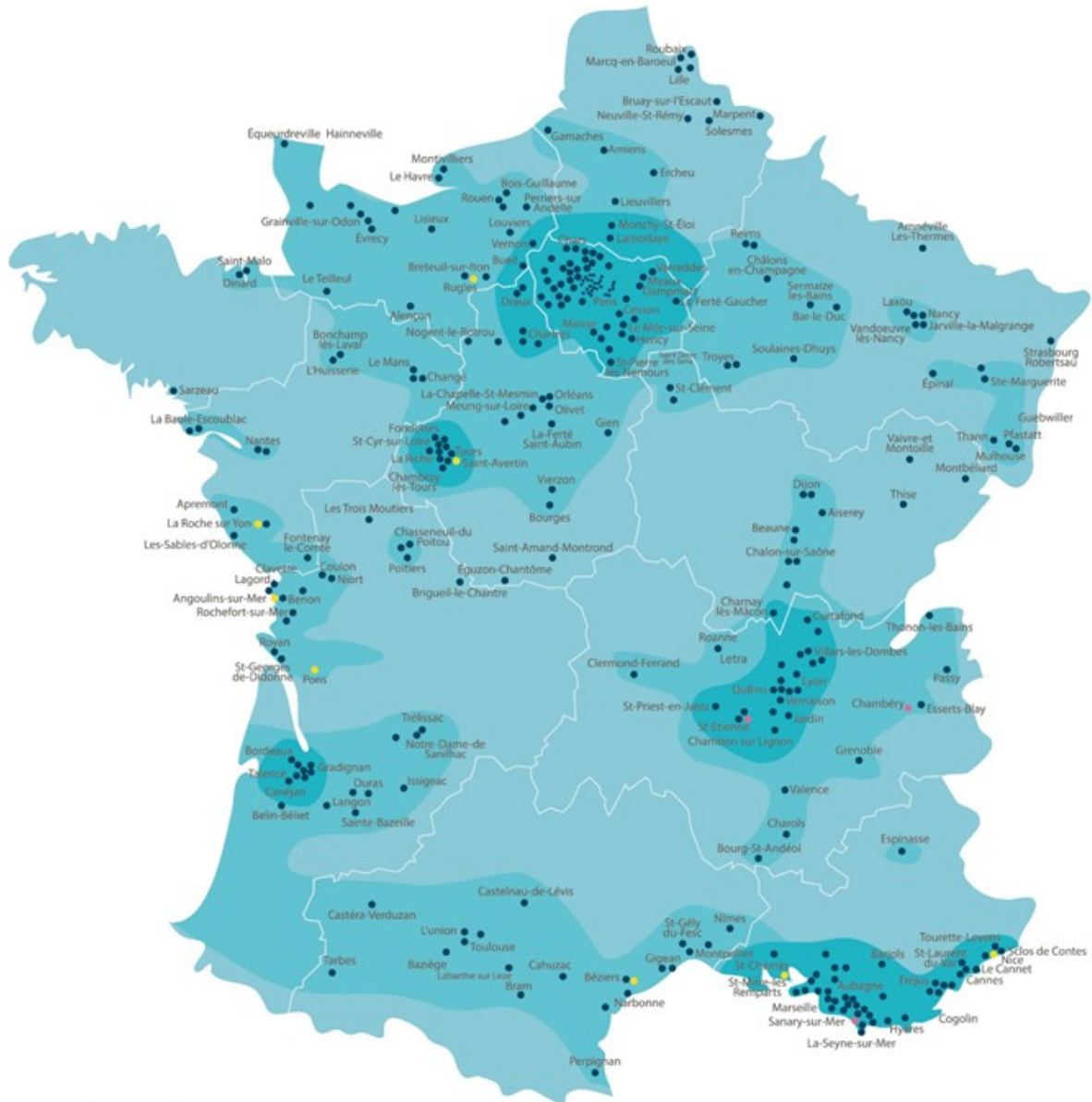

## THE KORIAN NURSING HOMES NETWORK IN FRANCE

Supplement: Multimedia Appendix 1 [file publichealth_v4i4e69_app1.pdf]
