# Supplementary material for: Issues in Building a Nursing Home Syndromic Surveillance System with Textmining: Longitudinal Observational Study
Source: JMIR Public Health Surveill. 2018 Dec 13;4(4):e69. doi: 10.2196/publichealth.9022 (PMC6315244; doi:10.2196/publichealth.9022)
Supplement: Multimedia Appendix 2 [file publichealth_v4i4e69_app2.pdf]

The anonymization process

We re-indexed all the residents' and NHs' index in order to protect personal privacy but will always be able to re-link them later by a trusted party if later needed. The new indexes are computed through piecewise linear increasing functions smoothed on the original indexes, functions which can be redefined periodically. Without an access on the Korian group original database it is then nearly impossible to find the matching between old and new indexes.
