# Supplementary material for: Issues in Building a Nursing Home Syndromic Surveillance System with Textmining: Longitudinal Observational Study
Source: JMIR Public Health Surveill. 2018 Dec 13;4(4):e69. doi: 10.2196/publichealth.9022 (PMC6315244; doi:10.2196/publichealth.9022)
Supplement: Multimedia Appendix 3 [file publichealth_v4i4e69_app3.pdf]

The BBV ARI-ILI and AGE syndromic information building process in four phases

Phase 1

Internal medical experts and PERMF users were interviewed on how to best define ARI-ILI and AGE syndromes with words [36]. Two lists were selected, one defining the first syndrome with words such as *acute*, *fever*, *flu*, *coughing*, *asthenia*, *pneumonia*, and a second with words such as *diarrhea*, *vomiting*, *with blood*, *abdominal pain*, *fever.* Among these words designed for the ARI-ILI syndrome, several of them describe non-specific respiratory diseases' symptoms. For example, *fever* and *coughing* can also come from other respiratory problems such as the Respiratory Syncytial Virus [50-51].

Phase 2

Phase 2 involved an algorithm of SQL queries automatically analyzing all health descriptions and picking the right sets of words describing each syndrome [52]. This data processing was then tried through small periods of winter, when there were numerous cases and through small periods of summer, when there were much less (especially for flu), analyzing every result, true positive (TP), true negative (TN) cases and each health description, to see what words to add and what words to remove to improve results. Each new iteration of this process resulted in better defined cases. Through this process the words' meaning and shape were also checked to exclude erroneous interpretations [55-56].

Phase 3

After this second step medical experts were asked again to check the syndromes, adding or removing some words to better describe them in a Delphi-like process [55-56] until reaching an agreement [57]. As in [58], the ARI_ILI and AGE syndromic definitions construction followed a process based on consensus and current use.

Phase 4

Finally, through whole past seasons, week after week, our syndromic numbers were compared with national trends defined elsewhere (*Sentinelles* network data [22]), the aim being to have concordant data and being able to use the CDC surveillance algorithms on them to detect or even better predict NH epidemics. Whenever an anomaly was found, the discrepancies periods were re-analyzed either to find an explication or to adjust the words' lists and/or combinations. For example, for the ARI-ILI syndrome, several wrong cases came from influenza and pneumonia vaccines. To conclude, all this process could be assimilated to supervised learning [59-60], defining the method with a small test sample and refining it with a bigger learning one, except that in this study, we had lots of data, enough data to test and learn on numerous samples, not fearing any over adjustment and not needing any cross-validation [61].

The four BBV tables

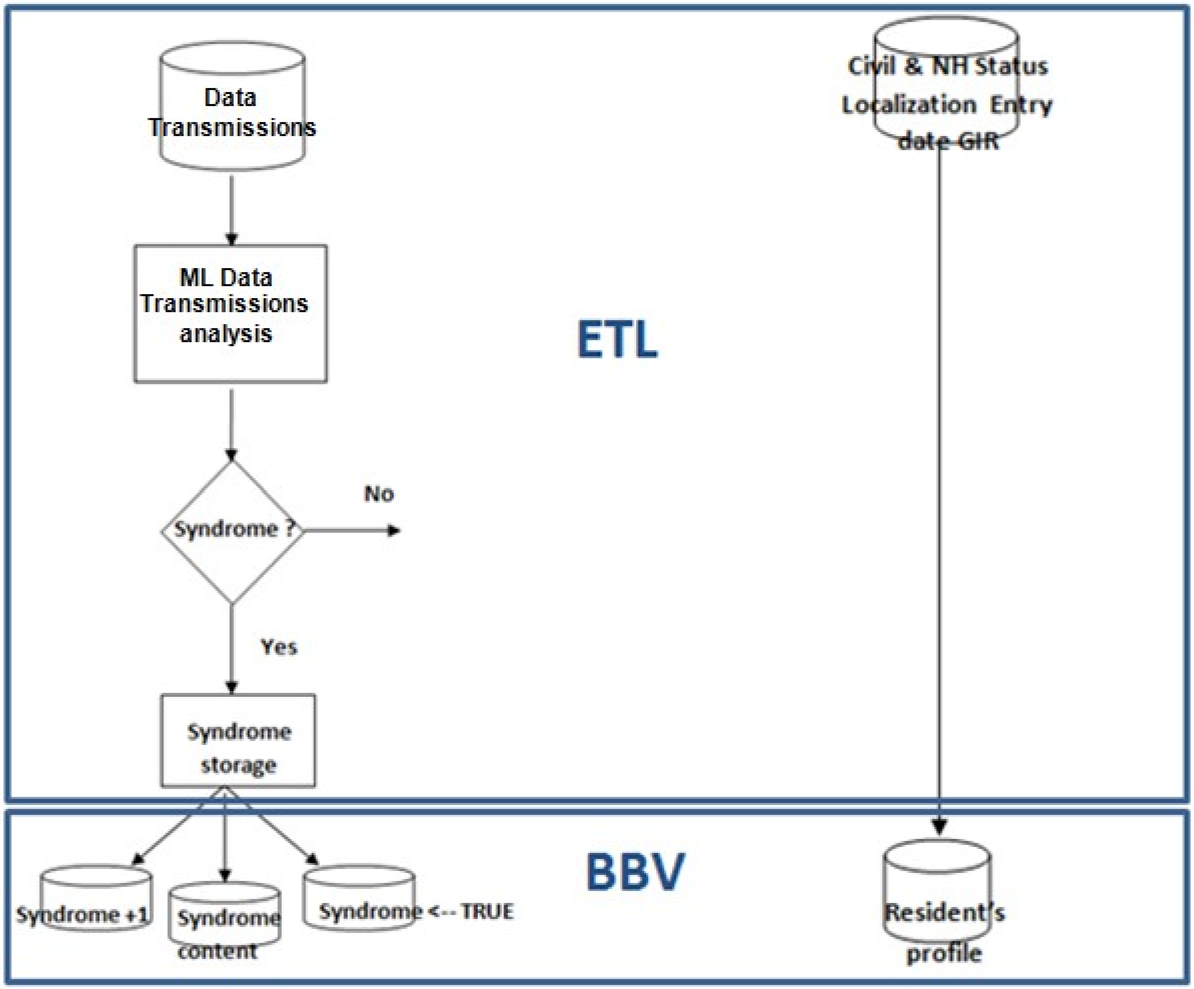

THE BBV SYNDROMIC INFORMATION SYSTEM BUILDING PROCESS

The whole syndromic building process is described on the left side of the figure.

Whenever machine learning data transmission analysis led to syndromic data and described one or several of the 26 syndromes (**Yes**), it was stored in three outcomes tables (bottom left of the figure). From left to right, the first table added one to each of the syndromes distributions needed to describe the data transmission, the second table recorded the syndromic sentence and the last one built the Boolean filters to use for subsequent SQL requests.

Otherwise, if data transmission analysis didn't lead to syndromic data (**No**), nothing was stored.

Example 1 of an ARI-ILI syndrome literal description and how it is processed:

"D: ASTHENIC ++ THIS MORNING. FEVERISH 101DEG. PALE, CONGESTED, WHEEZING. 1G PARACETAMOL GIVEN"

The first step involves analyzing this short sentence. The process catches the words *feverish, congested* and *wheezing* as describing an ARI-ILI syndrome. Then this ARI-ILI syndrome is stored in the BBV database through 3 different ways:

1- one is added to the NH's ARI-ILI syndromes' weekly count
2- the short sentence is fed in the literal syndromes' description table;

3- ARI-ILI syndrome is fed with TRUE and the 25 others fed with FALSE in the Boolean syndromes' description table.

Example 2 of a combined AGE / ARI-ILI syndrome literal description and how it is processed:

"ASTHENIC +++ VOMITING FOOD, FEVERISH RECORDING VITAL SIGNS : TA =11/7 , PULSE 97R, SAT 97%,TDEG;=102 PARACETAMOL SUPPOSITORY GIVEN AT 10:30PM TDEG=99 , SAT = 93 AT 4:30AM CHECK WITH MG MONITORING +++ (FLU SYNDROME ??)"

Here the process catches the words *vomiting* and *feverish* as describing an AGE-syndrome and the words *feverish* and *flu* as describing an ARI-ILI syndrome. Both syndromes are stored in the BBV database through 3 different ways:

1- one is added to the NH's AGE syndromes' weekly count;
2- one is added to the NH's ARI-ILI syndromes' weekly count;

3- the short sentence is fed in the literal syndromes' description table;

4- AGE and ARI-ILI syndromes are fed with TRUE and the 24 others fed with FALSE in the Boolean syndromes' description table.

With this method, syndromic data is split in 3 parts:

1- The twenty-six syndromes numbers traced every week in every nursing home, generating the syndromic surveillance tool (first table from the left);
2- The literal syndromic description of every impacted resident (second table from the left). These descriptions which sometimes directly refer to the resident or resident's relations are loaded separately with an exclusively restricted access.

3- The Boolean syndromic description of every impacted resident (third table on the left);

Finally, added to these syndromic data, we added the residents' profile with gender, age and Iso Resource Group at the NH entry and NH localization loaded on a fourth table (bottom right of the figure).

The BBV 26 syndromes list

Collected data covered elderly key concerns and health priorities. The complete twenty-six syndromes' list was:

1- ARI-ILI and AGE built through the 4 phases described above;
2- Hospitalizations and deaths directly built from the hospitalizations and deaths tables' extractions;

3- Twenty-two remaining syndromes built through the 3 phases described above as the fourth phase was not applicable to them: 1-pain, 2-behavior, 3-dementia, 4-general state alteration, 5-dehydration, 6-denutrition and swallowing, 7-cutaneous state, 8-allergies, 9-falls, 10-depression and dark thoughts, 11-cardio-vascular symptoms, 12-audition, 13-oral health, 14-cancer, 15-sleeping problems, 16-vaccination, 17-vision, 18-intestinal transit, 19-urinary track, 20-frailty, 21-overweight and 22-diabetes.
