# Supplementary material for: Issues in Building a Nursing Home Syndromic Surveillance System with Textmining: Longitudinal Observational Study
Source: JMIR Public Health Surveill. 2018 Dec 13;4(4):e69. doi: 10.2196/publichealth.9022 (PMC6315244; doi:10.2196/publichealth.9022)
Supplement: Multimedia Appendix 4 [file publichealth_v4i4e69_app4.pdf]

How public health alerts are defined:

The French public health agency triggers a public health alert after the following 3 steps are fulfilled: first, signals' reception (health events and/or statistical indicators), second, signals' validation after local, regional and national epidemiologists' verifications and third, assessing the health threat by public health authorities [70] (Santé Publique France SPF)

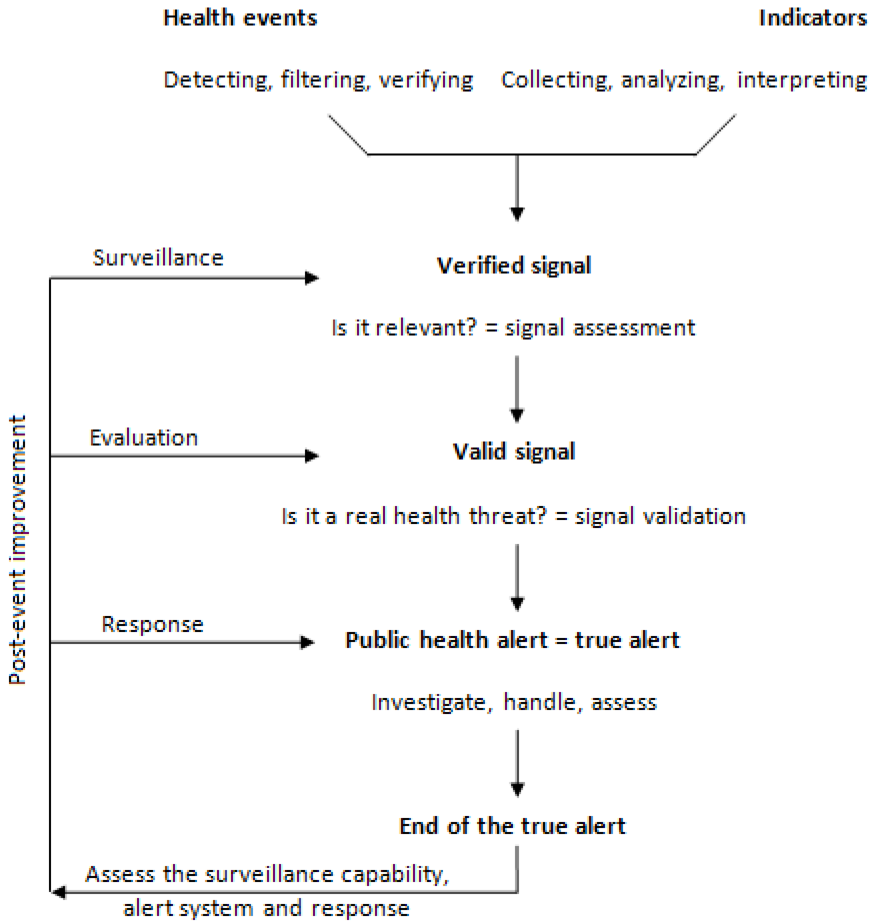

THE SURVEILLANCE, ALERT AND RESPONSE CONCEPTUAL FRAME PROCESS ACCORDING TO SANTÉ PUBLIQUE FRANCE [70]

For all surveillance methods, building time series (TS) and statistical indicators from them or from other available data is always the first step. Here in this experiment as reference material, we chose to use the Sentinelles network [22] outbreaks data.

Statistical alerts according to the Sentinelles network

Alerts are triggered whenever outbreak levels are exceeded. The outbreak detection level with the Sentinelles network corresponds to the estimated baseline prediction confidence interval upper-limit computed with the Serfling method [71-72]. For the ARI-ILI and AGE Sentinelles data, when weekly incidence crosses this threshold, there is a high probability of being in an epidemic period, especially when the weekly cases' numbers cross this threshold twice in the row [72].

True alerts according to the MASS system

The MASS system ((Module for the Analysis of SurSaUD and Sentinelles' data) combines 3 different statistical methods on 3 different data sources [69], including the Sentinelles network data to define its signals, verify and validate them. These outbreaks are defined by SPF following the whole process described in the figure above and are then **true, genuine alerts**.

How the surveillance® package works:

TS with the surveillance® package [67] involved defining them as disease progress objects containing two vectors: first, the observed number of weekly counts and second, a Boolean vector state indicating whether there was an outbreak that week or not [73]. Here in this experiment, we chose to use the Sentinelles outbreaks and so in the influenza case, the vector of outbreak states contained the ILI Sentinelles' epidemic weeks. Then we selected 4 algorithms to build our statistical indicators: Bayes and the 3 CDC CUSUM methods.

1- **The Bayes surveillance algorithm:** This algorithm, using the 12 former weeks, assumes that the reference values are identically and independently Poisson distributed where a Gamma-distribution is used as Prior distribution for the Poisson parameter. Within this framework, quantiles of the predictive posterior distribution are used as a measure for defining alarm thresholds and alarm weeks [12, 73].
2- **The 3 EARS' (early aberration reporting system) surveillance algorithms:** These algorithms, using 7, 9 or 12 preceding weeks [74-77] are non-historical methods based on positive 1-sided cumulative sums (CUSUM) calculations [74]. For C1 and C2, a warning is generated when the current count is greater than the baseline mean plus 3 standard deviations. For C3, a warning is generated when the last three CUSUMs' mean is greater than the baseline mean plus 2 standard deviations [75]. Each warning generates an alarm week. Further details can be found in [67] at the earsC section.

After these two steps, the signal is verified and the surveillance process starts as such. The last step is then comparing *alarm weeks* built with the *Bayes* or *EARS*_C_i, *i=1, 2, 3* algorithms to *epidemic weeks* (*outbreak weeks*, here *epidemic weeks* recognized as such by the *Sentinelles* network), the aim being the best possible overlapping between the two.
