# Supplementary material for: Issues in Building a Nursing Home Syndromic Surveillance System with Textmining: Longitudinal Observational Study
Source: JMIR Public Health Surveill. 2018 Dec 13;4(4):e69. doi: 10.2196/publichealth.9022 (PMC6315244; doi:10.2196/publichealth.9022)
Supplement: Multimedia Appendix 5 [file publichealth_v4i4e69_app5.pdf]

Syndromic Data-Flow Stability

Syndromic data-flow stability was defined here by 4 syndromes indexes: diabetes, cardio-vascular problem, depression and falls.

For the 2016 year, as 200 days-reference periods were needed, only residents with the first 222 (165+57 = 61% of 365) days of 2016 (denominators counts) could encounter these same syndromic events (numerators counts) through whole 200 days periods thereafter (reaching the end of February 2017: see figure below). To counter this, we then checked stability by comparing our four 2016 ratios to the 2015 ones, computing four 2015-2016 syndromic events relative ratios.

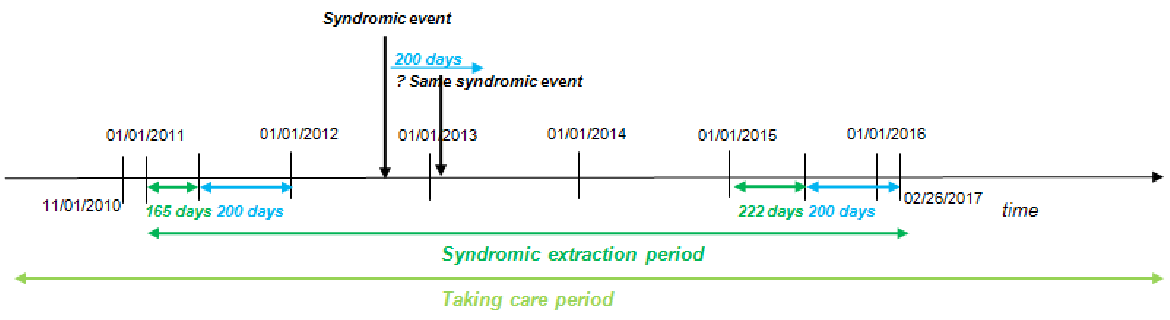

THE SYNDROMIC DATA FLOW STABILITY ASSESSMENT PROCESS

The 4 syndromes' data flow stability ratio (see table below) was computed as follows:

1- on denominator, for every year and every syndrome, the transmissions headcounts;
2- on numerator, the same syndromic events headcounts, for the same people on a 200 days period thereafter.

The small arrows under each attrition ratio showed the 2011-2015 trends. All four syndromic ratios through time were highlighted. The last column computed the 2015-2016 transmissions headcounts differences, allocated to 2015 transmissions headcounts and as explained above, showed some attrition. Every first transmission was followed on average by another one of the same type through a 200 days period for about 40% of diabetics, 50% of cardiac patients, 76% of depressives and 66% of frequent fallers for the 6 years period.

Syndrome headcounts, ratios & trends	2011	2012	2013	2014	2015	2016	2015-2016 Relative ratios
**Diabetes**	818/1574	765/1786	716/1736	724/1759	654/1658[a]	564/1496[a]	**162[a]/1658**
↘→	**52%**	**43%**	**41%**	**41%**	**39%**	**38%**	**10[b]%**
**Cardio-vascular**	1871/3682	2227/4533	2074/4223	2002/3893	1980/3953	1510/3327	470/3953
→	**51%**	**49%**	**49%**	**51%**	**50%**	**45%**	12%
**Depression**	6413/8254	5379/7073	4496/5900	4265/5407	4358/5458	3496/4623	862/5458
→	**78%**	**76%**	**76%**	**79%**	**80%**	**76%**	16%
**Frequent falls**	4458/6410	3942/6036	3249/5039	3190/4810	3126/4683	2746/4238	380/4683
↘→	**70%**	**65%**	**65%**	**66%**	**67%**	**65%**	8%

[a] 162 = 1658 – 1496
[b] 10% = 162/1658

Rating Syndromic Data Flow Stability During Time with Four Syndromes Frequencies: Diabetes, Cardio-vascular problems, Depression and Falls
